# The relationship of spectral sensitivity with growth and reproductive response in avian breeders (*Gallus gallus*)

**DOI:** 10.1038/srep19291

**Published:** 2016-01-14

**Authors:** Ye-Feng Yang, Jing-Song Jiang, Jin-Ming Pan, Yi-Bin Ying, Xiao-Shuang Wang, Ming-Li Zhang, Min-Si Lu, Xian-Hui Chen

**Affiliations:** 1 Department of Biosystems Engineering, Zhejiang University, Hangzhou 310058, China; 2 Zhejiang Guangda Breeding Poultry Corporation, Jiaxing 314423, China

## Abstract

A previous study demonstrated that birds that are exposed to light at night develop advanced reproductive systems. However, spectrum might also affect the photoperiodic response of birds. The present study was aimed to investigate the effects of spectral composition on the growth and reproductive physiology of female breeders, using pure light-emitting diode spectra. A total of 1,000 newly hatched female avian breeders (*Gallus gallus*) were equally allocated to white-, red-, yellow-, green- and blue-light treated groups. We found that blue-light treated birds had a greater and faster weight gain than did red- and yellow-light treated birds (*P* = 0.02 and 0.05). The red light expedited the sexual maturation of the chicks, whose age at sexual maturity was 7 and 14 days earlier than that of the green- and blue-light treated birds, respectively. The accumulative egg production of the red-light treated birds was 9 and 8 eggs more than that of the blue- and green-light treated birds. The peak lay rate of the red-light treated groups was significantly greater than the blue-light treated birds (*P* = 0.028). In conclusion, exposure to short-wavelength light appears to promote growth of female breeder birds, whereas exposure to long-wavelength light appears to accelerate reproductive performance.

The widespread use of artificial lighting to illuminate the night-time environment has raised significant concerns regarding organism health and ecology^1^,^2^. Despite significant increases in artificial light^3^, experimental studies of the effects of artificial light on organisms are not consistent, which is critical in order to avoid the confusing effects of variables such as metabolic disorders. Considering that avian breeders are exposed to artificial light throughout their lifespans, experimental studies on the effects of controlled light conditions on growth and reproduction in avian breeders are urgently needed.

Housing animals in a controlled light environment is useful for assessing the effects of light on animal models. An anatomical study indicated that the bird is one of the best studied animal groups with respect to the impact of artificial light^4^. It has been reported that birds exposed to artificial light at night develop their reproductive system up to one month earlier and moult earlier than birds maintained under dark nights^5^. Rowan^6^ hypothesized that constant light stimulates early breeding in London starlings (*Sturnus vulgaris*). Moreover, poultry scientists have documented that exposing birds to long days can stimulate reproduction outside the natural breeding period^7^. Furthermore, although the effects of constant light have begun to be elucidated^5^,^15^, knowledge about the growth and reproductive consequences of the light spectral composition is still limited. Nowadays, the spectra of light in urban areas is very diverse, resulting in a mosaic-like spatial distribution of different wavelengths of artificial light systems^8^. Although limited studies have been reported to investigate the effect of spectral composition on the growth and reproductive, the composition of the artificial light used in the studies are far from monochromatic^9^. For example, a previous study reported that green light does not affect the reproduction of birds during the laying phase^10^. However, another study indicated that green light consistently increased eggs laid than red light^11^. Another investigation demonstrated that testosterone is elevated in cockerels grown under green light, whereas the testes weights were heavier only in birds kept under white or red light^12^. The possible causes of these contradictory findings may be due to variability in the monochromatic light. The red, green and blue light used in those studies, which are generated by filters, are far from monochromatic. Therefore, research using filtered light might yield such confusing results. Indeed, one of the major challenges for experimental studies investigating the impact of artificial light on birds is the lack of commercial monochromatic light sources. However, a new monochromatic light source—the light-emitting diode (LED) lamp—is currently available commercially. It is a simple semiconductor device and consists of a single junction. Light is emitted when the junction is forward biased so that monitory carrier injection and electron-hole recombination occur. The major benefit of LED lamps is the single peak of light wavelength, which is characterized by a narrow half band output. Moreover, our previous study indicates that the LED light spectra can affect the photoperiodic response of birds^13^,^14^,^15^. Other investigations have demonstrated that the spectrum of light can affect growth (broilers)^16^, carcass characteristics (broilers)^17^, welfare (broilers)^18^, and behaviour (laying hens)^19^.

The hypothalamus is the central light conduit that feeds photic information to the growth and reproductive axes^20^. The ability of light to penetrate the hypothalamus of bird is dependent upon light wavelength^21^. Therefore, biological responses can be caused by various spectral sensitivities. The present study aim to investigate the effects of spectral composition on the growth and reproductive physiology of female breeders, using pure LED spectra.

## Results

### Body weight

**Figure 1:**
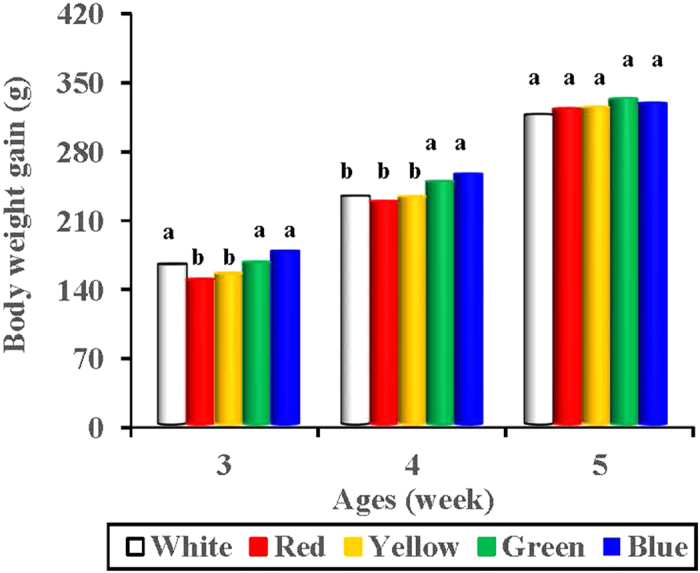
Body weight gain (g) in birds reared under different spectral composition. Each group of treated birds was exposed to either white light, red light, yellow light, green light or blue light. Body weight was individually measured at 3, 4 and 5 week of age, and the body weight gain was calculated. Data are expressed as the mean value ± SD. ^a,b^The mean values without a common superscript indicates statistically significant differences (*P* < 0.05).

We found that short-wavelength-light treated birds (green and blue light treatments) gained greater weight than did long-wavelength-light treated birds (red and yellow treatments) (*P* < 0.05; Fig. 1). A significant increase in body weight gain was observed in white-, green- and blue-light treated birds as early as 3 weeks of age compared with red- and yellow-light treated birds (*P* = 0.001 and 0.01, respectively). The trend continued to 4 weeks with the green- and blue-light treated birds. At 4 weeks, the green- and blue-light treated birds were even heavier than the white-light treated birds (*P* = 0.04 and 0.018, respectively). However, when the birds were grown to 5 weeks of age, the body weight gain differences between each light-treated group were eliminated, suggesting a compensatory growth of long-wavelength-light treated birds. At these ages, no difference was found in body weight either between green-light treated birds and blue-light treated birds (*P* = 0.604), or between red and yellow (*P* = 0.864).

Moreover, short-wavelength-light treated birds (green and blue light treatments) attained greater growth rates than did long-wavelength-light treated birds (red and yellow treatments) (*P* < 0.05) (data not shown). An increase in the relative cumulative growth rate was observed at 3 weeks of age in blue-, green and white-light treated birds compared with red- and yellow-light treated birds (*P* = 0.001 and 0.01, respectively). The relative cumulative growth rate of blue- and green-light treated birds was even greater than that of white-light treated birds at 4 weeks (*P* = 0.023 and 0.006, respectively). However, when grown to 5 weeks, no differences were observed between each light-treated group (*P* = 0.238).

### Sexual maturation

**Figure 2:**
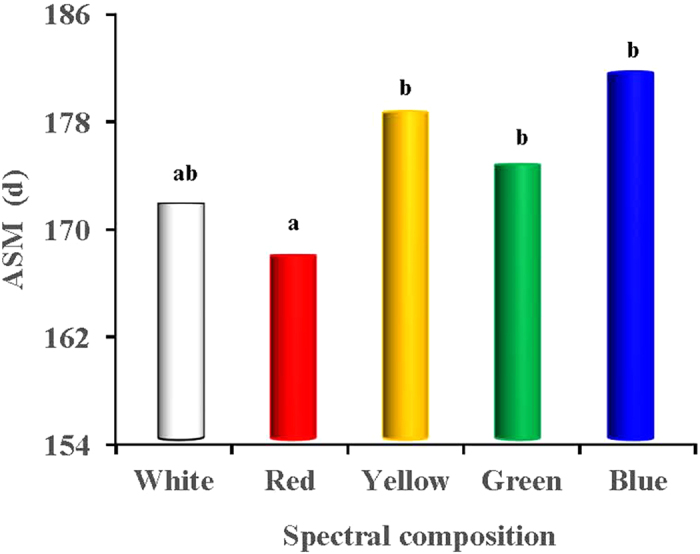
ASM (d) in birds reared under different spectral composition. Each group of treated birds was exposed to either white light, red light, yellow light, green light or blue light. Age at 50% rate of lay was defined as the age at sexual maturity (ASM).

**Figure 3:**
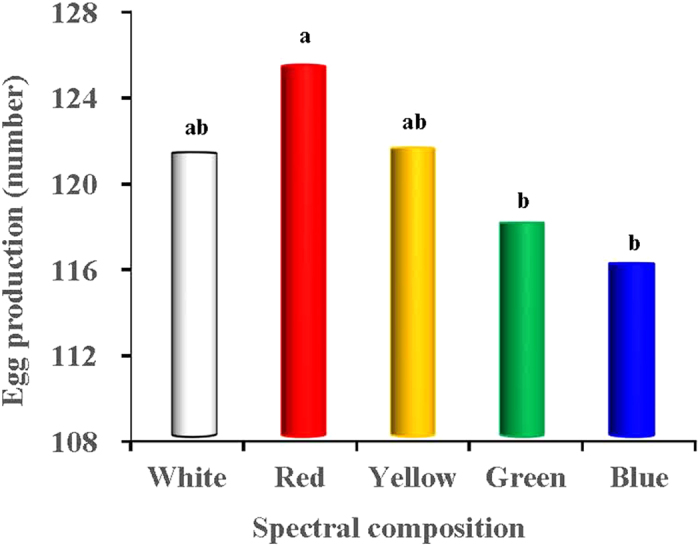
Accumulative egg production (n) per bird reared under different spectral composition. Each group of treated birds was exposed to either white light, red light, yellow light, green light or blue light. When the chicks grown to 22 week, most of them started to produce eggs. We measured the egg production of each light-treated group daily, then calculated egg production of each light treated-bird.

**Figure 4:**
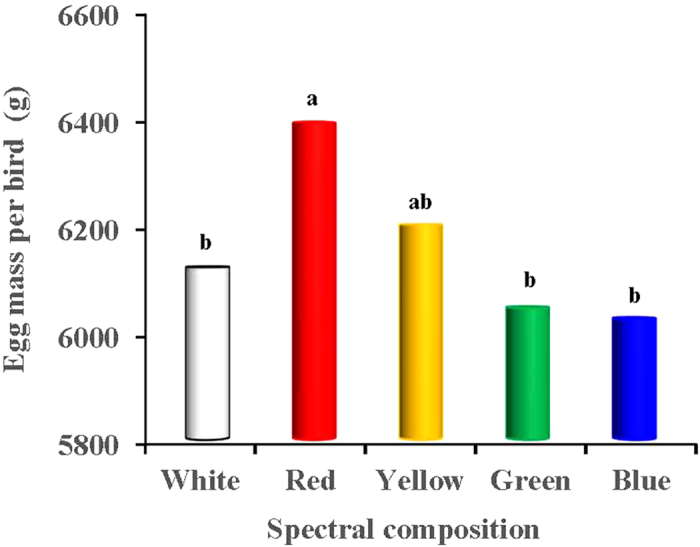
Accumulative egg mass (g) per bird reared under different spectral composition. Each group of treated birds was exposed to either white light, red light, yellow light, green light or blue light. When the chicks grown to 22 week, most of them started to produce eggs. Egg mass was obtained by calculating the cumulative average egg weight per bird per day of the total period (22–58 week).

We found that long-wavelength light expedited the age of sexual maturation (ASM) of the birds and promoted egg production, whereas short wavelength light delayed sexual maturation and reduced egg production (Figs 2, 3, 4). The red light expedited the sexual maturation of the birds, whose age at sexual maturity was 7 and 14 days earlier than that of the green- and blue-light treated birds (*P* = 0.018 and 0.003; Fig. 2), respectively. The greatest values of accumulative egg production and egg mass both occurred in the red-light treated birds (*P* < 0.05; Fig. 3). The egg production of the red-light treated birds was 9 and 8 eggs more than that of the blue- and green-light treated birds, respectively. Moreover, the total egg mass per bird of the red-light treated birds reached 6400 g, which was 375.4 g and 354.2 g more than that of blue- and green-light treated birds (*P* = 0.025 and 0.01; Fig. 4), respectively. The egg masses of the blue- and green-light treated birds were also lower than those of the yellow-light treated birds.

**Figure 5:**
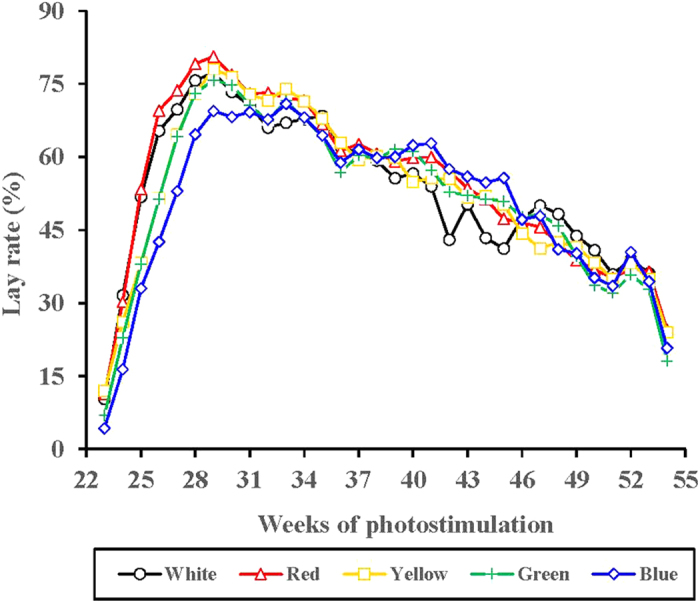
The lay rate of each light-treated group. When the birds grown to 22 weeks, most of them started to produce eggs. We measured the egg production of each light-treated group daily, then calculated lay rate of each light treated-group. Significant differences observed between red-treated group and yellow-treated group during 23–29 week. Statistics statement was not presented for the concern of clarity.

**Figure 6:**
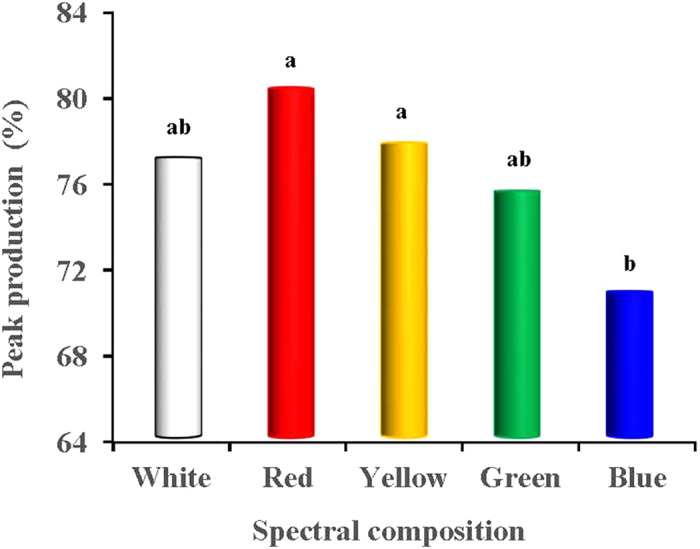
Peak lay rates (%) of each light-treated group. The peak lay rate of white-, red-, yellow- and green-treated groups were occurred at 29 week, whereas the peak lay rate of blue treated group was occurred at 33 week. Data are expressed as the mean value ± SD. ^a,b^The mean values without a common superscript indicates significant differences (*P* < 0.05).

**Figure 7:**
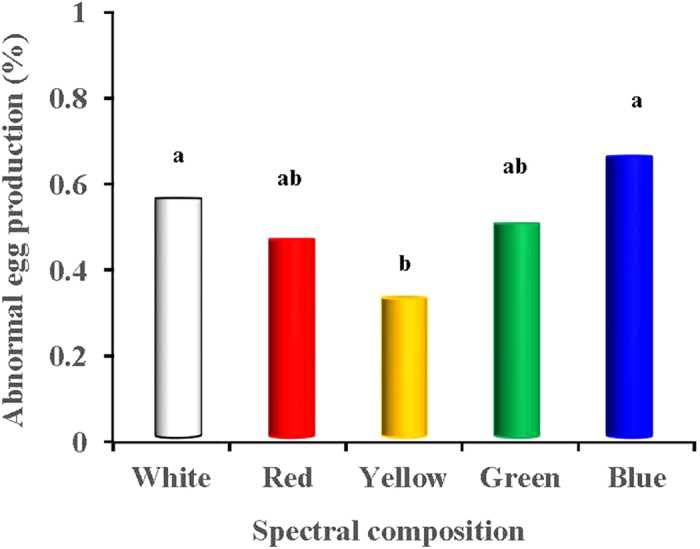
Abnormal egg production (%) of each light-treated group. We measured the abnormal egg (including deformity, crack, etc.) numbers daily, then calculated the abnormal egg rate. Data are expressed as the mean value ± SD. ^a,b^The mean values without a common superscript indicates significant differences (*P* < 0.05).

Figure 5 presents the weekly lay rates of the birds in each light-treated group. Variations in the weekly lay rate of the 5 light-treated groups was apparent at 23–29 weeks (*P* = 0.021). However, the weekly lay rate of the 6 light-treated groups was similar during the rest weeks (after 29 weeks). ANOVA analysis indicated that the average lay rate in the blue-light treated birds at 23–29 weeks was significantly reduced compared with the red-light treated birds (*P* = 0.004) (Fig. 5). However, although no significant differences in the average lay rate were observed between each light-treated group at 30–55 weeks, the long-wavelength light enhanced the weekly lay rate of the birds during most of the period compared with the short-wavelength-light treated groups. In addition, the peak production (lay rate) of the red-light treated birds were significantly greater than that of the blue-light treated birds (*P* = 0.028; Fig. 6). The rates of accumulation of abnormal eggs for each light-treated group are presented in Fig. 7. The accumulation of abnormal eggs was lowest in the yellow-treated group, which was significantly lower than the blue- and white-light treated groups (*P* = 0.008 and 0.037, respectively).

### Egg quality

We found significant effects of light composition on egg quality (Table 1). The egg weight in the green-light treated group was the greatest and was notably greater (*P* = 0.032) than that in the white-light treated group. The egg shape index of each light-treated group was similar (*P* = 0.226). The egg shell thickness of the white-light treated group was the lowest and that of the blue-light treated group was significantly increased (*P* = 0.314) compared with the red-, yellow- and white-light treated groups. In addition, the egg shell thickness values of the green- and yellow-light treated groups were increased (*P* = 0.226) compared with the white-light treated group. No significant differences in egg shell strength were observed among these light-light treated groups. The Haugh unit (Hu) of the eggs in the white-light treated group was the greatest.

### Fertility and hatchability

**Figure 8:**
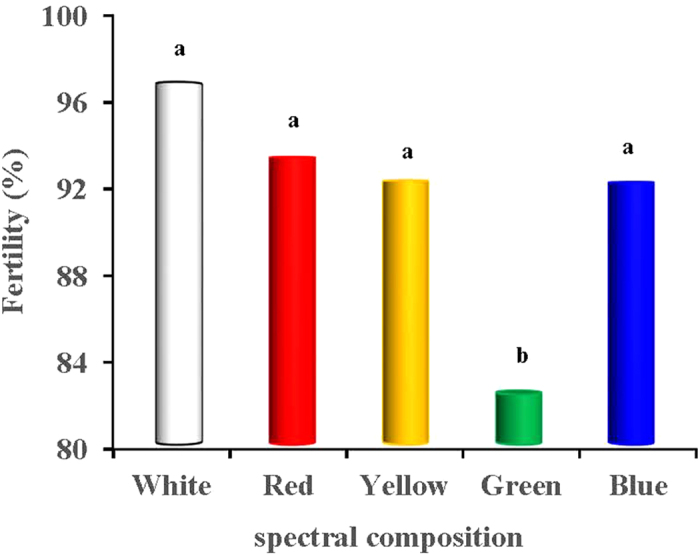
Fertility of eggs produced by birds exposed to different spectral composition. Each group of treated birds was exposed to either white light, red light, yellow light, green light or blue light. A total of 1,000 labelled eggs from 6 light-treated group were hatched in the same incubator under the controlled conditions of 37.5 °C and 55% relative humidity. These eggs were candled twice on the 7th and 14th days to remove unfertilized and dead-germ eggs to measure fertility. Data are expressed as the mean value ± SD. ^a,b^The mean values without a common superscript indicates significant differences (*P* < 0.05).

**Figure 9:**
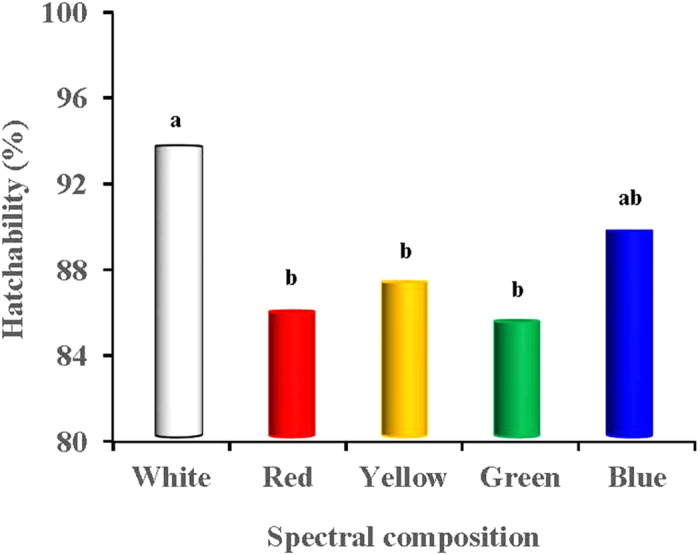
Hatchability of eggs produced by birds exposed to different spectral composition. The successfully hatched chicks of each group were recorded on the 21st day to determine the hatchability of the fertilized eggs.

Figure 8 showed that the fertility of the eggs produced in the green-light treated group was the lowest compared with the other light-treated groups (*P* = 0.002). The fertility in the green-light treated group was 14.71%, 11.18%, 10.08% and 10.00% lower than that in the white- red-, yellow- and blue-light treated groups (*P* = 0.016, 0.037, 0.041 and 0.041), respectively. However, no significant differences were observed among the white-, red-, yellow- and blue-light treated groups (*P* = 0.604). The hatchability of the fertile eggs produced in the white-light treated group was greater than that in the red-, yellow- and green-light treated groups (*P* = 0.039, 0.047 and 0.031; Fig. 9), respectively. In total, the hatchability of the white-light treated group was 7.96%, 6.49% and 8.42% greater than that observed in the red-, yellow- and green-light treated groups. However, no significant difference was observed between the hatchability of the white-light treated group and the blue-light treated group (*P* = 0.598).

## Discussion

The results of the present study confirm the idea of a stimulatory effect of spectral sensitivity light with various compositions on the growth and reproduction in female breeders. Our study highlights the fact that in female breeders, short-wavelength light promotes growth, whereas long-wavelength light improves reproductive performance.

We found that short-wavelength light treated birds gained more weight than long-wavelength-light treated birds. Green- and blue-light treated birds were significantly heavier than red- and yellow-light treated birds (3–4 weeks of age). Moreover, a significant increase in the relative cumulative growth rate was observed in the blue-, green- and white-light treated birds compared with the red- and yellow-light treated birds (3–4 weeks of age). Previous studies reported that red light promotes growth better than blue light in turkeys^22^. Similarly, some investigators have reported that the growth of red-light treated male and female turkeys is faster than that of blue-light treated turkeys^22^. However, other researchers have concluded that broilers^23^ and quails^24^ exposed to blue fluorescent lamps gain significantly more weight than those exposed to red fluorescent lamps. These contradictory conclusions probably may be due to the variety of light sources, because most of the previous commercial coloured lamps are inefficient in comparison with monochromatic light^25^. However, the spectrum of monochromatic light produced by LED light sources is specific and sufficiently narrow to enable the control of the wavelength within 2–5 nm in our study. In fact, electrophysiological and behavioural methods have confirmed that birds show different peak sensitivities to light spectra^26^. The peak wavelength of the blue and green LED light used in our study was 455 nm and 514 nm. Thus, the stimulatory effect of green and blue light on bird growth may due to the peak sensitivities. This finding is in accordance with previous studies on broiler chickens^27^,^28^, which demonstrates that broilers reared under blue or green light become significantly heavier than those reared under red light.

Our study revealed that long-wavelength light expedited the sexual maturation of birds and promoted egg production in the birds, whereas short-wavelength light delayed sexual maturation and reduced egg production. Red light expedited the sexual maturation of the birds, whose age at sexual maturity (ASM) was 7 and 14 days earlier than that of the green- and blue-light treated birds, respectively. The greatest values of accumulative egg production and egg mass both occurred in the red-light treated birds. The egg production of the red-light treated birds was 9 and 8 eggs more than that of the blue- and green-light treated birds, respectively. Moreover, in the present study, we found that the total egg mass per bird of the red-light treated birds reached 6400 g, which was 375.4 g and 354.2 g more than that of the blue- and green-light treated birds, respectively. Our results are consistent with the previous study by Gongruttananun^29^, who observed that the cumulative egg number was significantly increased in the red-light group during the early season, although the spectrum of red light did not affect the total egg production rate, compared with natural light. Indeed, the effect of red, white, blue and green light on the sexual maturation of the birds was consistent with previous general conclusions from studies of laying hens^30^. Moreover, Lewis, *et al.*^31^ previously demonstrated that green light does not enhance the performance of egg-type birds during the laying phase. In fact, the activation of retinal photoreceptors appears to be inhibitory to reproduction^32^. The response to visible radiation is probably mediated by the blue-green bands of the light spectrum (513–571 nm), in which the avian retina exhibits relative peak sensitivity^26^. Photostimulation of retinal photoreceptors by green light elevated green opsin gene expression, while non-detectable amounts of this gene were observed in the hypothalamus^33^, because the ability of green light to penetrate the tissue is poor^31^. In accordance with previous study^34^, we speculated that retinal photostimulation with green light might increase serotonin levels and inhibit reproductive performance, which was manifested by a considerable delay in egg production in green-light treated birds. Consequently, the photostimulation of retinal photoreceptors, which are sensitive to short-wavelength-light, appear to inhibit reproductive activity in birds^33^. On the other hand, the sensitivity of birds to long wavelength stimuli is a result of deep tissue penetration (hypothalamic extra-retinal photoreceptors), stimulating the reproductive axis^32^. The extra-retinal photoreceptors are located in the medial basal hypothalamus and lateral septal organ in the brain^35^. The information given by these reports may explain the greater egg production of red-light treated birds compared with green-light treated birds. However, the detailed mechanisms are still unclear.

In conclusion, we found that a pure spectral composition plays a vital role that affects the growth and reproductive physiology of female breeders. In addition, body weight gains of birds exposed to short wavelength, which are more sensitivity to birds, were greater than those of long wavelength treated birds. Finally, the present study indicates that red light expedites sexual maturation and promotes egg production in birds. The mechanisms through which the spectral composition affects the growth and reproductive response of birds are unclear.

## Material and Methods

All experimental protocols were approved by the committee of the Care and Use of Animals of the Zhejiang University. The methods were carried out in strict accordance with the guidelines of the Association for the Study of Animal Behaviour Use of the Zhejiang University.

### Animals and experimental design

The “Meihuang” female broiler breeder (*Gallus gallus*), a famous original yellow-feathered chicken in southern China, was selected for this study from the Zhejiang Guangda Breeding Poultry Corporation. This corporation is certified by the China Agricultural Ministry as one of the two national gene pools of native broiler breeders. A total of 1,000 female birds were equally divided into five light-treated groups (white, red, yellow, green and blue light-emitted dioxide [LED]) pipes in replicates. Each group of light-treated birds had the following similar initial body weights before treatment initiation: 30.5 ± 0.2 g (white), 30.7 ± 0.3 g (red), 30.5 ± 0.4 g (yellow), 30.6 ± 0.2 g (green), and 30.6 ± 0.1 g (blue).

During the brood phase (1 - 6 weeks), the birds were kept in small cages (1.4 m length × 1.4 m width × 0.4 m height) at a density of 25 birds/m^2^. After 6 weeks, the birds were transferred into the special tri-deck laying batteries. Each deck consisted of five cages (50 cm length × 38 cm width × 35 cm height) such that both sides of one laying battery reared 90 birds (2 × 15 cages, 3 birds per cage). The birds had *ad libitum* access to feed and water. The environmental parameters in the house were set at 32.5 °C (temperature) and 67.5% (relative humidity) during the brooding period and at 22.8 °C (temperature) and 62.5% (relative humidity) after the brooding period.

**Figure 10:**
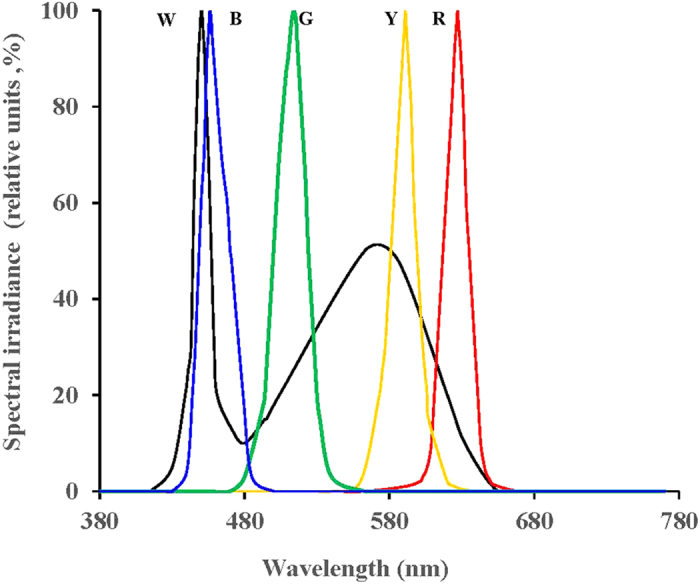
Relative spectral irradiance of the LED pipes used: W = White, R = Red, Y = Yellow, G = Green, B = Blue.

Five types of 60-cm-long LED pipes were used to provide the following five spectral compositions: red (618.4–620.8 nm), yellow (587.9–589.1 nm), green (516.0–521.0 nm), blue (455.0–461.0 nm) and white (400–760 nm) (Fig. 10). The light schedule followed the recommendation from the Breeding Corporation, which is 8 h daily (8 L : 16 D; lights off at 1500 h) before 20 weeks and 16 h daily (16 L : 8 D; lights off at 2300 h) after 20 weeks (during this period, the birds begin to produce eggs). The light intensity of the LED groups was controlled by adjusting the pulse width modulation (PWM) values. The light intensities were controlled according to the average value measured from 12 points within the cage, including the four middle points between the five cages of each deck.

All of the light sources were equalized at the same light intensity of 40 lux. The light intensity was measured using a Digital Lux Meter (AR823, Digital Lux Meter Co. Ltd., China). The light intensity was not adjusted according to the spectral sensitivity. During the brood phase, the light sources were attached to the cage ceiling, and the light intensity was controlled according to the average value measured from five points within the cage, including the center and four corners of the cage floor. During the growing and laying phases, the light sources were hung at 1.5 m above the top of ladder-shaped laying batteries.

### Data acquisition

Growth performance was measured as body weight gain (g) and relative growth rate (%). During the brooding period, the body weights of the birds exposed to each light treatment were measured. Then, the body weight gain and relative growth rate were calculated. Reproductive physiology was measured as egg production (n), egg quality, sexual maturity, hatchability and fertility. When grown to 21 weeks (laying period), the birds began to produce eggs. During the laying period, egg production and bird numbers were recorded daily. The lay rate was calculated daily and averaged weekly. The age at a 50% lay rate was defined as the age at sexual maturity (ASM). Abnormal eggs (including deformities and cracks) were also recorded daily. Artificial insemination was performed at 36 weeks of age by skilled veterinarians. Because reproduction is the most important responsibility of female breeders, fertility and hatchability should be used as the prerequisite indicators in evaluations of the reproductive physiology of female broiler breeders^25^. To eliminate the effect of sex ratio on fertility^36^, artificial insemination was used to detect fertility and hatchability in our study. The semen was obtained from male breeders of the same batch. After artificial insemination, one hundred eligible eggs were randomly selected from each light group during the following two days. A total of 1,000 labelled eggs were hatched in the same incubator under controlled conditions of 37.5 °C and 55% relative humidity. These eggs were candled twice on the 7th and 14th days to remove unfertilized and dead-germ eggs and to measure fertility. The successfully hatched birds in each group were recorded on the 21st day to determine the hatchability of the fertilized eggs. The egg quality was also measured. Thirty intraday eggs of each light group were randomly selected for assessments of weight, shape index, shell thickness, shell strength and Haugh unit (Hu).

### Statistical analysis

Data were subjected to statistical analyses using SPSS Statistical software (V. 20.). Statistical analysis of data was factorial by rooms and by light. Rooms were found not to be significant for all treated variables, and results were retested by one-way. ANOVA was performed to analyze the effects of light spectral composition on birds. Homogeneity of variance was checked for each set of data, and no transformations were applied. When appropriate, post hoc comparisons were made by using least significant differences. Data are presented as the mean ± SD. In every case, difference between group means and correlation coefficient were considered statistically significant if the value of *P* < 0.05.

## Additional Information

**How to cite this article**: Yang, Y.-F. *et al.* The relationship of spectral sensitivity with growth and reproductive response in avian breeders (*Gallus gallus*). *Sci. Rep.* **6**, 19291; doi: 10.1038/srep19291 (2016).

## Figures and Tables

**Table 1:** The egg quality of each light-treated group (44 week).

Light treatments	Egg weight (g)	shape index	Egg shell thickness, mm	Haugh unit	Egg shell strength (N)
White	51.02 ± 0.82^b^	1.30 ± 0.01^b^	0.28 ± 0.01^c^	84.73 ± 1.38^a^	37.69 ± 1.19^a^
Red	52.55 ± 0.78^ab^	1.31 ± 0.01^b^	0.29 ± 0.01^bc^	81.29 ± 1.83^ab^	33.99 ± 1.22^a^
Yellow	52.77 ± 0.79^ab^	1.30 ± 0.01^b^	0.29 ± 0.00^b^	80.38 ± 1.67^ab^	35.38 ± 0.88^a^
Green	53.88 ± 0.88^a^	1.31 ± 0.01^b^	0.30 ± 0.01^ab^	80.70 ± 1.66^ab^	35.96 ± 0.99^a^
Blue	53.35 ± 0.88^ab^	1.30 ± 0.01^b^	0.31 ± 0.01^a^	81.24 ± 1.73^ab^	35.38 ± 1.28^a^

Values are described as the mean values ± SD.^a,b,c^The mean values in a column without a common superscript exhibited significant differences (*P* < 0.05).
